# The alternative splicing factor hnRNP A1 is up-regulated during virus-infected epithelial cell differentiation and binds the human papillomavirus type 16 late regulatory element

**DOI:** 10.1016/j.virusres.2007.09.006

**Published:** 2008-02

**Authors:** Thanaporn Cheunim, Jingxin Zhang, Steven G. Milligan, Maria G. McPhillips, Sheila V. Graham

**Affiliations:** Institute of Biomedical and Life Sciences, University of Glasgow, Glasgow G12 8TA, Scotland, UK

**Keywords:** HPV16, human papillomavirus type 16, ESS, exonic splicing suppressor, LRE, late regulatory element, 3′ UTR, 3′ untranslated region, EMSA, electrophoretic mobility shift assay, SR proteins, serine/arginine-rich proteins, GST, glutathione *S*-transferase, RBD, RNA-binding domain, SF2/ASF, splicing factor 2/alternative splicing factor, hnRNP A1, heterogeneous ribonucleoprotein particle A1, U1snRNP, U1 small nuclear ribonucleoprotein particle, NHK, normal human keratinocytes, CAT, chloramphenicol acetyltransferase, HPV16, hnRNP A1, RNA processing, Alternative splicing

## Abstract

Human papillomavirus type 16 (HPV16) infects anogenital epithelia and is the etiological agent of cervical cancer. We showed previously that HPV16 infection regulates the key splicing/alternative splicing factor SF2/ASF and that virus late transcripts are extensively alternatively spliced. hnRNP A1 is the antagonistic counterpart of SF2/ASF in alternative splicing. We show here that hnRNP A1 is also up-regulated during differentiation of virus-infected epithelial cells in monolayer and organotypic raft culture. Taken together with our previous data on SF2/ASF, this comprises the first report of HPV-mediated regulation of expression of two functionally related cellular proteins during epithelial differentiation. Further, using electrophoretic mobility shift assays and UV crosslinking we demonstrate that hnRNP A1 binds the HPV16 late regulatory element (LRE) in differentiated HPV16 infected cells. The LRE has been shown to be important in temporally controlling virus late gene expression during epithelial differentiation. We suggest that increased levels of these cellular RNA processing factors facilitate appropriate alternative splicing necessary for production of virus late transcripts in differentiated epithelial cells.

## 1 Introduction

Human papillomaviruses (HPVs) are small circular double-stranded DNA viruses that infect both cutaneous and mucosal epithelia (Howley, 1996). HPV infection commonly causes benign papillomas or warts. A significant subset of HPVs infects the anogenital tract. These can be divided into “low risk” and “high risk types”. The latter types can cause lesions that, on some rare occasions, develop to malignancy. Often, integration of high-risk HPV DNA into host genomes is an essential first step in the tumourigenesis pathway but this integration event abrogates completion of the normal virus life cycle. HPV16 is one of the most abundant high-risk types associated with approximately 60% of cervical cancer cases worldwide (zur Hausen, 1996).

During a normal infection, transcription of the 7.9 kb HPV16 genome yields polycistronic RNAs, which are processed by alternative splicing to produce mature messenger RNAs (Milligan et al., 2007). The virus genome can be sub-divided into three parts, an early region containing six early genes (E1–E7), a late region with two late genes (L1 and L2), and a long control region (LCR). The HPV life cycle is completely dependent upon epithelial differentiation. The six early proteins, which are mainly involved in episomal maintenance of the virus genome, transcriptional regulation, and cell transformation, are produced throughout the virus life cycle (zur Hausen, 1996). In contrast, expression of the virus capsid proteins, L1 and L2 is restricted to terminally differentiated epithelial cells. Restriction of production of the highly immunogenic capsid proteins to these cells may allow the virus to complete its life cycle undetected by the host immune response (Stoler et al., 1989; Stubenrauch and Laimins, 1999).

*Cis*-acting regulatory elements have been identified in the coding regions and the late 3′ untranslated regions (UTRs) of several papillomaviruses (Graham, 2006). It is proposed that these elements employ different RNA-based mechanisms to regulate virus late gene expression. Two HPV16 coding sequence elements have been suggested to regulate splice site selection. The first, in the E4 open reading frame acts as a splicing enhancer to facilitate early mRNA processing and inhibit late gene expression (Rush et al., 2005). The 5′ end of the L1 open reading frame harbours a number of regulatory sequences stretching over 514 nucleotides (Collier et al., 2002) that are proposed to inhibit nuclear RNA processing. There is good evidence that the core of this element binds heterogeneous nuclear ribonucleoprotein particle (hnRNP) A1 and it acts as a splicing silencer, at least in HeLa cells, to suppress the use of the splice acceptor site located at the 5′ end of the L1 open reading frame (Zhao et al., 2004). Inhibitory elements also exist in the L2 coding region of HPV16. The L2 elements are poorly defined but bind hnRNP K and poly(rC)-binding proteins 1 and 2 and may regulate translation (Collier et al., 1998).

The best characterised 3′ UTR regulatory element is the bovine papillomavirus type 1 (BPV1) inhibitory element, which is located in the BPV1 long control region. It is 53 nucleotides in length and contains a good consensus 5′ splice site. This 5′ splice donor site base-pairs with the 5′ end of U1 snRNA, allowing U1 snRNP to assemble on the element (Furth and Baker, 1991; Furth et al., 1994). A direct consequence of this binding is that U1 70 K, one subunit of U1 snRNP, interacts with poly(A) polymerase to inhibit polyadenylation (Gunderson et al., 1998). It is thought that this mechanism is employed to inhibit virus late gene expression in undifferentiated, BPV1 infected cells, but it remains unclear how such suppression is released to allow late gene expression in terminally differentiated cells.

**Fig. 1:**
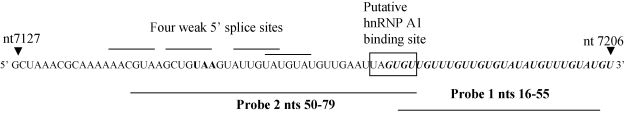
RNA sequence of the HPV16 LRE at the junction of the L1 open reading frame and the long control region (LCR). The L1 UAA stop codon is in bold type. The four weak 5′ splice sites (Cumming et al., 2003) are underlined and the 3′ GU-rich region is in italic type. A putative hnRNP A1 binding site is boxed. Truncated LRE probes used in experiments described in Fig. 5 are indicated beneath the sequence.

A similar element is present in the HPV16 late 3′ UTR. This 79-nt late regulatory element (LRE) overlaps the 3′ end of L1 gene and extends into the late 3′ UTR (Kennedy et al., 1990). The element can be divided into two parts, a 5′ LRE portion, that contains four weak 5′ splice sites, and a 3′ LRE portion that is GU-rich (Fig. 1). Site-directed mutation analysis revealed that the entire sequence is required for the repressive function of the element on gene expression in undifferentiated epithelial cells (Cumming et al., 2003). A U1 snRNP-like complex also binds this element (Cumming et al., 2003). In addition, it can also bind the auxiliary splicing factor U2AF (Dietrich-Goetz et al., 1997), the key SR protein SF2/ASF (McPhillips et al., 2004), the polyadenylation factor CstF64, and the elav-like shuttling protein HuR (Koffa et al., 2000) suggesting that the element regulates virus late gene expression by more than one post-transcriptional pathway.

If these elements regulate viral RNA processing during the virus life cycle then the proteins they bind may be regulated during epithelial differentiation. So far only SF2/ASF levels have been shown to respond to virus infection and epithelial differentiation (McPhillips et al., 2004). Increased levels of SF2/ASF may facilitate the extensive splicing and alternative splicing of virus late transcripts that is required in virus-infected, differentiated epithelial cells (Milligan et al., 2007). The counterpart of SF2/ASF in splicing regulation is hnRNP A1 (Pinol-Roma, 1997). While SF2/ASF binds exonic sequence enhancers to promote splicing of nearby introns, hnRNP A1 binds exonic sequence silencers to inhibit such splicing (Sanford et al., 2005). There is evidence for regulation of alternative splicing patterns by these two proteins in a concentration-dependent manner (Eperon et al., 2000). Studies in mice suggested that hnRNP A1 and SF2/ASF expression varies among tissues and different proportions of the two proteins were also observed (Hanamura et al., 1998; Kamma et al., 1995). In addition to regulating splicing, hnRNP A1 has been reported to be involved in mRNA export (Izaurralde et al., 1997; Mili et al., 2001), mRNA stability (Hamilton et al., 1997) and translation (Bonnal et al., 2005).

In this study, we demonstrate a significant specific increase in expression of nuclear hnRNP A1 during differentiation of HPV16 genome-containing epithelial cells in both monolayer and organotypic raft culture. The timing and level of increase is similar to that of SF2/ASF, the counterpart of hnRNP A1 in splicing regulation. Further, we demonstrate that hnRNP A1 binds the HPV16 LRE in the nucleus of differentiated W12 cells and GST-hnRNP A1 can bind the element directly *in vitro*. HPV16-induced changes in expression of both hnRNP A1 and SF2/ASF may facilitate alternative splicing of virus late transcripts.

## 2 Experimental

### 2.1 Cell culture

The 20863 (W12E) cell line is a subclone of the W12 line, an HPV16 positive cell line derived from a low-grade cervical lesion (Stanley et al., 1989), which contains 50–100 episomal copies of HPV16 genomes (Jeon et al., 1995). The HaCaT cell line is a spontaneously immortalised aneuploid human keratinocyte line, which is capable of differentiation (Boukamp et al., 1988; Schoop et al., 1999). For monolayer culture, W12E cells were co-cultured with mitomycin C-treated J2 3T3 fibroblast feeder cells at a ratio of 1:5, seeding W12E cells at 2 × 10^5^ cells/100 mm dish (McPhillips et al., 2004). The W12E line was grown for up to 5 days for undifferentiated cells, and 10 days for differentiated cells, respectively. The HaCaT cells were seeded at similar density but grew more rapidly and so were grown for 3 days for undifferentiated cells, and 6 days for differentiated cells. All cell lines were cultured in DMEM supplemented with 10% foetal calf serum, 2 mM glutamine, cholera toxin (8.4 ng/ml), hydrocortisone (0.4 μg/ml), and epidermal growth factor (10 ng/ml).

### 2.2 Nuclear and cytoplasmic extraction

For W12 cells, feeder cells were removed by treatment with 0.1% trypsin–0.5 mM EDTA prior to utilising for extraction. Nuclear and cytoplasmic extracts were prepared as described (Dignam et al., 1983). In some cases, HeLa nuclear extracts were purchased from 4C Biotech, Seneffe, Belgium.

### 2.3 Western blotting

Proteins were separated on 12% SDS-PAGE before electroblotting onto polyvinylidene fluoride (PVDF) membranes (Amersham). The membranes were blocked in 5% (w/v) dried milk powder in phosphate-buffered saline (PBS) overnight at 4 °C. Primary antibodies were diluted in PBS with 5% (w/v) dried milk powder, 0.05% (v/v) Tween-20 (PBS-T) and incubated with the membranes for 1 h at room temperature with shaking. The anti-hnRNP A1 antibody, 4B10 (gift from Gideon Dreyfuss, University of Pennsylvania, USA), was used at a dilution of 1/500, monoclonal antibodies against SF2/ASF (Mab 96; Zymed), involucrin (SY5; Sigma), keratin 10 (K8.60; Sigma), filaggrin (PRB-417P; CRP Inc.) and GAPDH (6CS; Biodesign International) were used at a dilution of 1/1000. After washing with PBS-T, the membranes were incubated with an anti-mouse secondary antibody–horseradish peroxidase (Sigma) at a dilution of 1/1000 in PBS-T with 5% (w/v) dried milk powder for 1 h at room temperature. Following washing in PBS-T, antibody–protein complexes were visualised using ECL reagents (Amersham) according to the manufacturer's instructions.

### 2.4 Electrophoretic mobility shift assay (EMSA)

Primer sequences used to generate riboprobe templates by PCR amplification of pCATPE445 (Cumming et al., 2003) are shown in Table 1. PCR products were purified on 6% polyacrylamide gels. Using *in vitro* transcription, riboprobes were synthesised with the Stratagene RNA transcription kit with addition of 25 μCi [α-^32^P]UTP (800 mCi/mmol; NEN) following the manufacturer's protocol. The full-length riboprobes were purified from a 5% denaturing polyacrylamide gel. EMSAs were carried out as described (Black et al., 1998). For competition EMSAs, specific competitor RNA was *in vitro* transcribed with the addition of unlabeled UTP substituting for [α-^32^P] UTP. Non-specific competitor RNA was transcribed from pBluescript KS(+) (Stratagene) polylinker linearised with EcoRI. Supershift EMSA experiments were carried out by preincubation of nuclear extracts with the anti-hnRNP A1 antibody for 30 min on ice. The EMSA reactions were electrophoresed on a 5% non-denaturing gel.

### 2.5 GST-hnRNP A1 protein purification

GST-tagged hnRNP A1 expression plasmid (pGEX-hnRNP A1; gift from Ron Hay, University of Dundee, UK) was transformed into BL-21 cells with induction of protein expression by adding 250 μl IPTG (100 mg/ml) to the cell culture. Transformed cells were lysed in 1 ml lysozyme (10 mg/ml) on ice for 15 min before incubating with 100 μl Triton X-100 for another 15 min. The supernatant was collected by spinning at 9500 rpm and mixed with 1 ml of 50% glutathione sepharose (Amersham) for 1 h before washing with PBS. The protein was eluted in 5 mM reduced glutathione in 50 mM Tris pH 8.0 three times and the concentration of the purified hnRNP A1 was determined by Bradford assay.

### 2.6 Immunohistochemistry and immunofluorescence of raft tissues

Organotypic raft culture was carried out as described (Meyers et al., 1992). Briefly, to establish a collagen matrix (2.5–3.0 ml/raft), 6.25 × 10^5^ J2 3T3 cells were mixed with 80% collagen type IV, 10% reconstitution buffer (2.2% (w/v) NaHCO_3_, 4.77% (w/v) HEPES in 0.062N NaOH), 10% 10× DMEM, and 0.024N NaOH. The collagen mixture was left to solidify in a 6-well cell culture plate before seeding 1.0 × 10^6^ epithelial cells suspended in E-medium (Meyers and Laimins, 1994) onto the collagen matrix surface. After incubating overnight at 37 °C to allow cells to attach to the surface of the collagen gel, and before adding E-medium, the raft system was transferred to a wire grid to bring the system up to the liquid–air interface. The rafts were fed with fresh E-medium containing C8:0 (10 μM) on alternate days for 12 days.

Organotypic raft tissues were fixed in 10% neutral-buffered formalin overnight, paraffin-embedded, cut into 4 μm cross sections, and placed on poly-l-lysine (Sigma) coated slides. Immunohistochemistry was carried out using the ABC Elite kit (Vector Laboratories) according to the manufacturer's instruction. Briefly, sections were dewaxed by incubating in xylene and rehydrated in a graded series of alcohol (100%, 100%, 95%, 75%, and 50% ethanol). Antigen retrieval was achieved by heating in citrate buffer (1.5 mM citric acid, 8.0 mM Tri-sodium citrate) for 15 min before blocking the sections with normal blocking buffer for 30 min. Monoclonal antibody was added for 1 h at room temperature. Diaminobenzidine tetrahydrochloride (DAB) (Vector Laboratories) was used as the chromagen to visualise the sections. Monoclonal antibodies against involucrin (SY5; Sigma; 1/1000) and hnRNP A1 (4B10; 1/1000) were used. For immunofluorescence, after being dewaxed and rehydrated, the sections were blocked in PBS-20% calf serum for 1 h before incubating with primary antibody for 1 h at 4 °C. Following washing with PBS, the sections were incubated with FITC conjugated secondary antibody for 1 h and mounted in VECTASHIELD mounting medium with DAPI (Vector Laboratories), and visualised using a Zeiss LSM510 laser scanning confocal microscope.

## 3 Results

### 3.1 hnRNP A1 expression is up-regulated during HPV16 infected epithelial cell differentiation

**Fig. 2:**
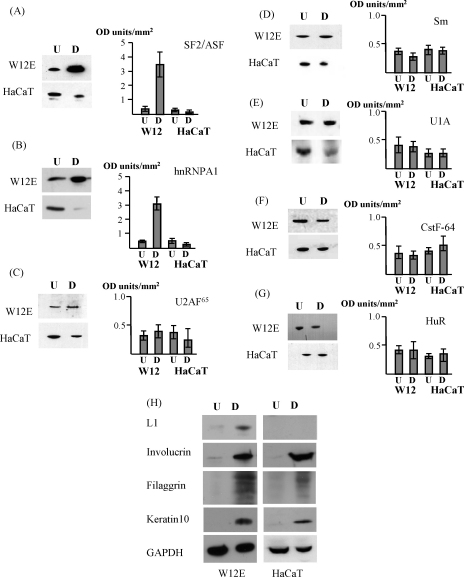
hnRNP A1 is up-regulated during differentiation of W12E cells. Cell lysates were prepared from undifferentiated (U) and differentiated (D) W12E and HaCaT cells. 10 μg of each extract was fractionated on SDS-PAGE and Western blotted with antibodies against the proteins indicated beside the graphs associated with each set of Western blots. (A) SF2/ASF (B) hnRNP A1 (C) U2AF^65^ (D) Sm protein (E) U1A (F) CstF-64 (G) HuR. Graphs show quantification of the Western blot data by densitometry scanning and show the mean and standard deviation from the mean of three to five separate experiments. The scale on the *Y*-axis in (A) and (B) is different to the scale for (C–G). (H) Western blots of undifferentiated and differentiated W12 and HaCaT cells probed with antibodies against involucrin, keratin 10 and filaggrin to demonstrate extent of differentiation of the cells. W12 cell extracts were probed with an anti-L1 antibody to demonstrate production of virus late proteins, associated with terminally differentiated, virus-infected epithelial cells. Blots were reprobed with an antibody against GAPDH as a loading control.

**Fig. 3:**
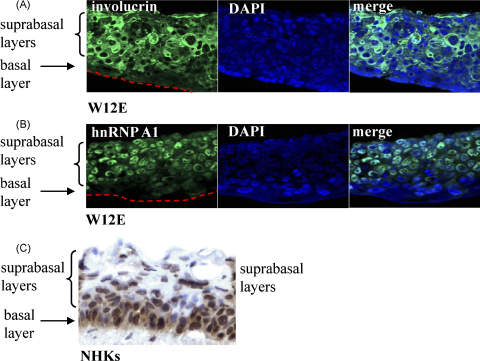
Immunostaining of organotypic raft culture sections demonstrating epithelial differentiation stage-specific and HPV16-mediated regulation of hnRNP A1. 4 μm raft tissue sections stained with (A) anti-involucrin antibody or (B) anti-hnRNP A1 antibody. Nuclei are stained with DAPI. The basal and suprabasal layers of the epithelia are indicated. The dotted red line indicates the junction of the raft tissue and the collagen layer. (C) Immunohistochemistry staining for hnRNP A1 (brown colour) of a 4 μm section of normal human foreskin keratinocytes. Nuclei are counterstained with eosin (blue colour). The basal and suprabasal layers of the epithelium are indicated.

We reported previously that the key splicing regulator SF2/ASF is up-regulated during differentiation of HPV16 infected epithelial cells. The antagonistic counterpart of SF2/ASF in regulating alternative splicing is hnRNP A1 (Eperon et al., 2000). HPV16 late transcripts are extensively alternatively spliced (Milligan et al., 2007) so we examined the relative levels of hnRNP A1 and SF2/ASF during epithelial differentiation using the W12 cell line. W12 cervical epithelial cells are immortal, but not transformed, differentiate in monolayer and organotypic raft culture and harbour episomal copies of the HPV16 genome (Jeon et al., 1995). Upon differentiation W12 cells express markers of differentiated epithelial cells and begin to express virus late proteins (McPhillips et al., 2004; Milligan et al., 2007) making them a good model system for the infectious life cycle of HPV16 in cervical epithelial cells. In addition, we carried out a similar analysis in HaCaT cells. The immortalised HaCaT human keratinocyte cell line contains no virus genomes (Boukamp et al., 1988; Schoop et al., 1999) but, similar to the W12 line, is able to differentiate in monolayer and organotypic raft culture (McPhillips et al., 2004). We used HaCaT cells rather than normal human keratinocytes (NHKs) because in monolayer culture, in our hands, HaCaT cells differentiate to a greater extent. In addition, we chose HaCaT cells over NHKs to allow comparison of levels of proteins in two established cell lines rather than in primary cells versus established cell lines. Any such comparison has pitfalls so in the quantification analyses W12 and HaCaT cells are dealt with separately. In Fig. 2 levels of expression of individual proteins in each line are expressed relative to levels of expression of the housekeeping protein GAPDH in undifferentiated or differentiated cells. Differentiation of W12 and HaCaT cells was demonstrated by expression of involucrin and keratin 10, both markers of differentiated suprabasal epithelial cells, and filaggrin, a marker of granular layer cells (Fig. 2H). For filaggrin, bands from 30 to 20 kDa were observed in the differentiated cells. This ladder of bands results from degradation of the protein, an event observed in late stages of epithelial differentiation (Fuchs, 1990). Expression of filaggrin demonstrated that a good level of epithelial differentiation was achieved in W12 cells and HaCaT cells although levels of the protein were not as high in the latter. In addition, expression of the major capsid protein, L1, in differentiated W12 cells confirmed that virus late gene expression was achieved. Besides SF2/ASF and hnRNP A1, levels of expression of a range of RNA processing factors that had previously been suggested to regulate HPV late gene expression were examined by Western blotting (Dietrich-Goetz et al., 1997; Cumming et al., 2003; McPhillips et al., 2004). Expression of hnRNP A1 was up-regulated in differentiated W12E cells (Fig. 2B) while in differentiated HaCaT cells a down-regulation was observed compared to undifferentiated cells. Quantification of results from five Western blot experiments indicated that there was a four to eight-fold up-regulation of hnRNP A1 during W12E differentiation (Fig. 2B) similar to that found for SF2/ASF (McPhillips et al., 2004). In contrast, there was around a two-fold decrease in hnRNP A1 levels in differentiated, compared to undifferentiated HaCaT cells. It is clear that HaCaT cells do not differentiate to the same extent as W12 cells. So final levels of hnRNP A1 in fully differentiated normal keratinocytes may be even smaller than that observed in HaCaT cells suggesting an even greater difference in levels of the protein in differentiated virus-infected versus non-virus-infected cells. We confirmed this decrease in hnRNP A1 expression during differentiation of normal human keratinocytes grown in organotypic raft culture (Fig. 3C). No significant up-regulation of other RNA processing proteins, whether splicing related (Fig. 2, C–E) or not (Fig. 2Fand G), was observed (note the changed *Y*-axis scale) demonstrating that levels of SF2/ASF and hnRNP A1 were specifically increased in differentiated W12 cells.

In case the changes in hnRNP A1 levels we observed were specific to differentiation in monolayer culture, we carried out immunofluorescence studies in W12E organotypic raft culture tissue. Involucrin expression commenced in the cell cytoplasm of the suprabasal layers as expected, indicating that these cells were differentiated (Fig. 3A). Similarly, increased levels of hnRNP A1 were clearly observed in the suprabasal layers of the raft tissue but the protein showed strong nuclear staining, with some cytoplasmic staining as expected (Fig. 3B). Compared to W12E tissue, raft tissue derived from normal human keratinocytes (Fig. 3C) showed staining with anti hnRNP A1 antibody in immunohistochemistry mainly in the basal layer and a decreased level of staining in the suprabasal layers. This confirms our Western blot data in monolayer cells. Immunohistochemistry using antibodies against SF2/ASF (manuscript in preparation), HuR (manuscript in revision), U1A and U2AF^65^ confirmed the results of the remaining Western data (data not shown).

### 3.2 The HPV16 LRE interacts with hnRNP A1

**Fig. 4:**
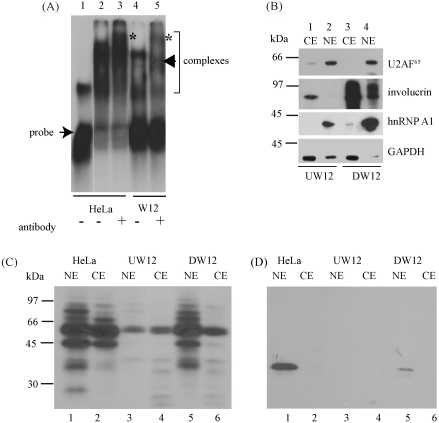
hnRNP A1 binds the HPV16 LRE. (A) Electrophoretic mobility shift assay showing interaction of hnRNP A1 and LRE RNA in HeLa and W12 cells. Free probe and the RNA/protein complexes formed are indicated. Asterisks and an arrowhead indicate RNA/protein complexes that have been supershifted due to binding of the anti-hnRNP A1 antibody in tracks 3 and 5. Track 1, [α-^32^P] rUTP-labelled LRE RNA probe alone (the LRE RNA is a stem loop structure (Cumming et al., 2003); the upper band is due to secondary structure formation); track 2, LRE RNA probe incubated with HeLa nuclear extract; track 3, as in track 2 but nuclear extract preincubated with anti-hnRNP A1 antibody; track 4, LRE RNA probe incubated with W12E nuclear extract; track 5, as in track 4 but nuclear extract preincubated with anti-hnRNP A1 antibody. (B) Western blot of nuclear (tracks 2 and 4) and cytoplasmic (tracks 1 and 3) extracts from undifferentiated (tracks 1 and 2) and differentiated (tracks 3 and 4) W12E cells showing fractionation of U2AF^65^ and hnRNP A1 mainly with the nuclear fraction and involucrin mainly with the cytoplasmic fraction as expected. The increase in levels of involucrin in tracks 3 and 4 confirms that the W12 cells have differentiated. (C) Autoradiogram of an SDS-PAGE fractionation of UV crosslinking of [α-^32^P] rUTP-labelled LRE RNA probe with nuclear (NE) and cytoplasmic (CE) fractions of HeLa and undifferentiated (U) and differentiated (D) W12E cells. (D) Western blot of the SDS-PAGE in (C) probed with anti-hnRNP A1 antibody.

hnRNP A1 has been shown to bind an exonic sequence silencer in the 5′ portion of the L1 open reading frame (Zhao et al., 2004). However, the HPV16 LRE that regulates late gene expression also contains a potential hnRNP A1 binding site (Fig. 1) (Burd and Dreyfuss, 1994), and if it bound hnRNP A1, this complex could be part of, or compete with, the LRE-binding SF2/ASF-containing complex (McPhillips et al., 2004). We determined whether the LRE interacts with hnRNP A1 using EMSA and UV crosslinking studies. Fig. 4A, tracks 2 and 4, shows that complexes formed when radiolabelled LRE RNA was incubated with HeLa and W12 nuclear extracts. The binding pattern in W12E cells was compared to that in HeLa cells because HeLa extracts are most frequently used in the literature to demonstrate binding of proteins to RNA (e.g. Burd and Dreyfuss, 1994). When the extracts were preincubated with anti hnRNP A1 antibody (tracks 3 and 5) supershifted bands were observed (starred bands) indicating that hnRNP A1 is a component of at least one of the complexes. In track 3 a band (starred) can be observed with lower mobility than the uppermost band in track 2 indicating that the antibody has reacted with the RNA/protein complex and retarded its mobility. In track 5 mobility of the upper band has been retarded (starred) but a supershift has also been induced in the lower band (arrowhead) indicating that there may be two different hnRNP A1-containing RNA/protein complexes that can form upon the LRE in W12 extracts. Next UV-crosslinking studies were used to confirm the EMSA data. Fig. 4C shows that at least seven proteins were UV-crosslinked to the LRE from HeLa cell extracts (Fig. 4C, tracks 1 and 2) and from differentiated W12 cell extracts (tracks 5 and 6). In contrast only one major band was detected with undifferentiated W12 cell extracts (tracks 3 and 4) although the same amount of protein extract was used in each crosslinking reaction. In HeLa and differentiated W12 cells more proteins were bound in nuclear extract than in cytoplasmic extract. Fig. 4D shows that when the gel was Western blotted and probed with an anti-hnRNP A1 antibody, bands were detected in tracks 1 and 5. Binding was most efficient in the presence of nuclear extract where the protein is most abundant (Fig. 4B) as expected. This indicates that hnRNP A1 can bind the LRE in W12 cells in a differentiation stage-specific manner. Fig. 4B demonstrates differentiation of the W12 cells and efficient fractionation of the nuclear and cytoplasmic compartments and confirms up-regulation of hnRNP A1 in differentiated W12 cells. GAPDH RNA levels vary during epithelial differentiation with a decrease during differentiation (Steele et al., 2002). We have observed a consistent small decrease in levels of GAPDH protein (Fig. 2H) upon differentiation and this may account for the reduction in levels of the protein in the nuclear fraction observed in Figure 4B, track 4.

### 3.3 hnRNP A1 directly interacts with the LRE

**Fig. 5:**
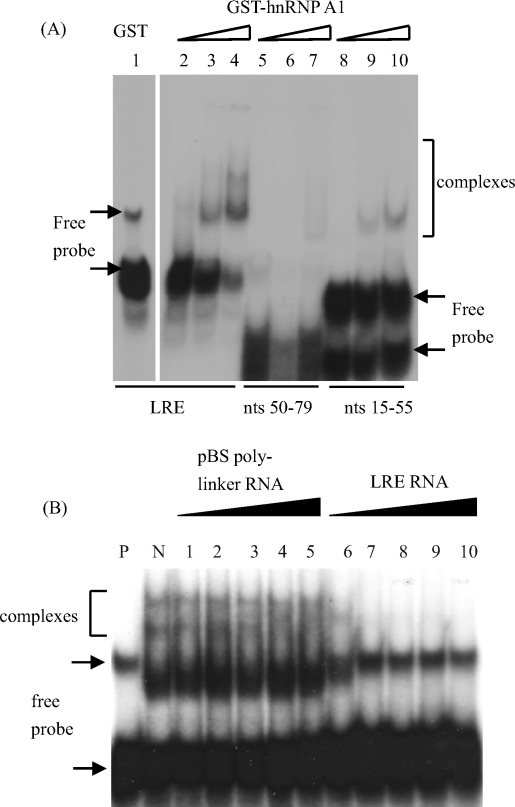
Nuclear hnRNP A1 binds the LRE directly. (A) Electrophoretic mobility shift analysis of binding of GST (500 nM) and GST-hnRNP A1 (45, 90, 180 nM) to [α-^32^P] rUTP-labelled LRE or truncated LRE RNA probes. The probes used are shown in Fig. 1 and indicated below the autoradiograph. Arrows indicate free probe. A bracket indicates RNA/protein complexes. (B) Competition electrophoretic mobility shift experiment using a non-specific competitor, unlabelled *in vitro*-transcribed pBluescript polylinker RNA (70 nts) (pBS) (lanes 1–5) or a specific competitor, unlabelled *in vitro* transcribed LRE RNA (79 nts) (lanes 6–10). P; [α-^32^P] rUTP-labelled LRE probe alone, N; probe plus nuclear extract. Both competitors were added to reactions at 1, 2, 4, 8 and 16-fold molar excess. Arrows indicate free probe. A bracket indicates protein/RNA complexes.

To discover whether hnRNP A1 could interact directly with the LRE, GST-hnRNP A1 was expressed and purified from *E. coli* and then incubated with [α-^32^P]-labelled full-length LRE probe in an EMSA experiment. Direct binding of hnRNP A1 to the LRE began to be observed at 90 nM (Fig. 5A, track 3) with an increase in binding at 180 nM (Fig. 5A, track 4). A corresponding, very significant decrease in levels of free probe could be seen in this track as expected. Molar amounts of probe and protein were calculated such that the probe was always in excess. This allows observation of increasing complex formation with increasing protein concentration. However, free probe is never entirely depleted. GST alone did not bind the LRE (Fig. 5A track 1). Free probe migrated as two bands because the LRE sequence has the potential to form significant secondary structure (Cumming et al., 2003). Fig. 5A (tracks 5–7) shows that the short 3′ LRE probe that is U-rich but does not contain the entire putative hnRNP A1 binding site did not bind the protein efficiently because little complex formation was observed. In contrast, a region of the LRE containing the four weak 5′ splice sites and the putative hnRNP A1 binding site bound the protein because some complex formation could be seen. However, complex formation was weak indicating that this probe did not bind as well as the entire LRE sequence (Fig. 5A, tracks 6–10). This indicates that the entire LRE sequence is required for efficient binding of hnRNP A1.

To demonstrate that hnRNP A1 binds specifically to the LRE, competition EMSA experiments were performed. GST-hnRNP A1 was incubated with [α-^32^P]-UTP-labelled LRE in the presence of non-specific or specific competitors (Fig. 5B). Inhibition of complex formation occurred when cold LRE probe, a specific competitor, was added to the reaction mixture at one-fold molar excess (Fig. 5B, tracks 1–5). When the cold competitor RNA is in significant molar excess it can compete with the labelled RNA probe for binding hnRNP A1. In contrast, the non-specific competitor, pBS polylinker RNA, could not interrupt complex formation when incubated with the probe from 1- to 16-fold molar excess. This is because the non-specific competitor RNA does not have any binding sites for hnRNP A1. The data clearly show that hnRNP A1 binds specifically to LRE-RNA.

## 4 Discussion

Using monolayer and organotypic raft culture systems, we observed up-regulation of hnRNP A1 during differentiation of W12E cells, which contain episomal copies of the HPV16 genome and provide a good model for the life cycle of HPV16 in cervical epithelial cells. It is known that factors involved in gene regulation are often up-regulated during tumour progression. This is not the reason for our observations here. First, the W12E line that we have used is capable of producing HPV16 capsid proteins (Fig. 2H) and thus of completing the virus life cycle. Life cycle completion and tumourigenesis are thought to be mutually exclusive events for HPVs. Second, when raft cultures of a subclone of the W12 cell line, W12G, where the virus genome is integrated into the host genome (one of the first steps in HPV-associated tumour progression) were examined a diffuse uniform distribution of the protein was observed (data not shown). The pattern of up-regulation of expression of hnRNP A1 is similar to what we observed previously for SF2/ASF (McPhillips et al., 2004). This regulation is specific for these splicing factors because Western blot and immunostaining of raft culture section with antibodies against a range of other RNA processing factors showed no such regulation (Fig. 2 and data not shown). The decrease in hnRNP A1 (and other RNA processing proteins) levels in differentiated HaCaT cells and NHKs is expected. Differentiated epithelial cells begin to shut off nuclear functions and in the final stages of differentiation, in the uppermost epithelial layers, the nuclei disintegrate. The difference in hnRNP A1 protein expression patterns between non-virus-infected cells (HaCaT and NHK) and virus-infected cells (W12E) implies that the presence of HPV16 genomes in the infectious state (episomal genomes, capable of completing the virus life cycle) in W12E cells caused these changes in levels of hnRNP A1. hnRNP A1 and SF2/ASF cooperate to define exons and introns and to regulate alternative splicing by binding to exonic splicing enhancers or exonic splicing silencer, respectively (Caceres et al., 1994). This is the first report of regulation of two functionally related cellular proteins by HPV infection in an epithelial differentiation-specific manner. SF2/ASF expression is regulated by HPV16 E2 (McPhillips et al., 2004; Mole and Graham, manuscript in preparation). However, preliminary data indicates that E2 may not regulate expression of hnRNP A1 (data not shown) and we are currently investigating the mechanisms upregulating this protein during differentiation of virus-infected epithelia.

It has been proposed that changes in the relative levels of SF2/ASF and hnRNP A1 may be important in regulating splice site usage (Hanamura et al., 1998; Kamma et al., 1995). It is clear that these proteins respond in a similar manner to virus infection during epithelial differentiation and this is suggestive of a functional link. Early in the virus life cycle there may be little need to splice the polycistronic transcripts produced from the virus genome (Wildridge and Graham, unpublished data). The major splice events that do occur are in the E6/E7 region of the genome where splicing from nt 226 to 409 is important (Zheng et al., 2004). These 5′ and 3′ splice sites conform to good consensus sequence and may not require high levels of SF2/ASF and hnRNP A1 to regulate their use. mRNAs produced late in the life cycle utilise a good consensus 5′ splice site at nt 880 in the E1 open reading frame but this is spliced to one of two possible 3′ splice sites; nt 3357 in the E2 open reading frame and nt 5637 in the L2 open reading frame, both of which deviate somewhat from the consensus sequence (Milligan et al., 2007), and may require a combination of SF2/ASF and hnRNP A1 binding to cognate sites in the E4 (Rush et al., 2005) and L1 exons (Zhao et al., 2004) to aid their utilisation. Increasing levels of hnRNP A1 and SF2/ASF in cells would aid this alternative splicing. These proteins are key regulators of alternative splicing of the *tat* gene of HIV-1 through competition for binding to a dual binding site in exon 3 (Zhu et al., 2001). *In silico* analysis of the L1 gene revealed a number of other potential hnRNP A1 binding site (data not shown) as well as several SF2/ASF binding sites but none of these overlap. Although both proteins bind the LRE, SF2/ASF does not bind directly (McPhillips et al., 2004) meaning that they are unlikely to compete for binding. hnRNP A1 appears to bind cooperatively to exons in a 3′ to 5′ direction and such binding can be blocked by SF2/ASF (Zhu et al., 2001; Damgaard et al., 2002; Marchand et al., 2002). Perhaps hnRNP A1 is recruited to the LRE and aids further loading onto the L1 exon in competition with SR proteins. Finally, the distance between the LRE and the L1 coding region hnRNP A1 binding sites does not preclude their cooperation: although the cooperative mechanism has not been worked out as yet, it is clear that hnRNP A1 binding sites need not be adjacent in exons and can indeed be some distance apart (Blanchette and Chabot, 1999). In addition, one of the key late events in the virus life cycle is amplification of virus genomes from 50–100 copies to over 1000 copies, which may all be transcriptionally active. Up-regulation of SF2/ASF and hnRNP A1 could facilitate in a general manner efficient and accurate production of virus late mRNAs as both these proteins are important in the early stages of splicing (Reed, 2000).

The data presented here demonstrate that hnRNP A1 may interact directly and specifically with the HPV16 LRE. In differentiated W12 cell extracts at least one major LRE/protein complex is formed that contains hnRNP A1. This complex is in the nucleus and so is likely involved in nuclear RNA processing. Analysis of the LRE sequence revealed one good candidate hnRNP A1 binding site (UUAGUGU) (Burd and Dreyfuss, 1994), located in the middle of the LRE (Fig. 1, Table 2). However, use of deletion mutations of the LRE in UV crosslinking revealed that no simple mutation abrogated hnRNP A1 binding suggesting that several motifs within the sequence cooperate to allow interaction with hnRNP A1 (data not shown). EMSA demonstrated that binding to the U-rich 3′ LRE was weak but binding to the 5′ region containing four 5′ splice sites and the putative hnRNP A1 binding site was stronger, but not as strong as binding to the entire LRE. GST-hnRNP A1 binding experiments indicated that the LRE bound hnRNP A1 with medium affinity according to Selex analysis of hnRNP A1 high affinity binding sites (Burd and Dreyfuss, 1994). Interestingly, in Selex the winning sequence was very similar to consensus vertebrate 5′ and 3′ splice sites. Thus, it is possible that the four weak 5′ splice sites in the LRE together with the putative hnRNP A1 binding site could help recruit hnRNP A1. In addition, it is known that hnRNP proteins bind U-rich sequences promiscuously so some binding to the 3′ LRE is expected. Whether hnRNP A1 binds the LRE as part of the splicing-like complex that the element also binds (Cumming et al., 2003), or indeed competes with this complex for binding to the LRE, remains to be tested.

hnRNP A1 has been demonstrated to bind a *cis*-acting RNA sequence in the HPV16 L1 coding region in HeLa cells transfected with an expression construct for the entire HPV16 coding region (Zhao et al., 2004). This binding suppressed the use of the 3′ splice site located immediately upstream of the L1 AUG, resulting in inhibition of splicing from the major 5′ splice site in the early region to the L1 3′ splice site. Thus, splicing of any L1 transcripts synthesised in undifferentiated virus-infected cells would be inhibited. This fits with our observation of the presence of unprocessed, nonpolyadenylated late transcripts in undifferentiated W12 cells (Milligan et al., 2007). It is possible that the HPV16 LRE could cooperate with the L1 coding region binding site to inhibit HPV16 late gene expression. However, in the experiments in HeLa cells the LRE had to be deleted from the HPV16 genome to achieve efficient late gene expression. This implies that the LRE may have a more important role than the L1 coding region regulatory sequence in repressing late gene expression in undifferentiated epithelial cells. Further, since we have shown that levels of hnRNP A1 are low in undifferentiated W12 cells it is currently unclear whether hnRNP A1 suppression of late region splicing occurs in a real infection.

## 5 Conclusions

We have demonstrated an increase in hnRNP A1 levels in HPV16 infected, differentiated epithelial cells, which parallels that seen for SF2/ASF (McPhillips et al., 2004). Binding of hnRNP A1 to the LRE in the late 3′ UTR may regulate RNA processing. However, hnRNP A1 can have positive as well as negative effects on gene expression (Blanchette and Chabot, 1999) and can act on cellular processes, both nuclear and cytoplasmic other than splicing regulation (Krecic and Swanson, 1999). A combination of different regulatory events is likely in regulation of HPV16 late gene expression.

## Figures and Tables

**Table 1:** Primer sequences and annealing positions in the HPV16 genome for generation of templates for UV crosslinking and EMSA RNA probes

Primer name	Promoter	Genomic location (nts)	Sequences
NRE probe forward	T3	7128–7144	5′ CAGAGATGCAATTAACCCTCACTAAAGGGGCTAAACGCAAAAAACG 3′
NRE probe reverse	None	7186–7207	5′ CTGAGCTCACATACAAACATATACACAAC 3′
NRE 15–55 forward	T3	7142–7163	5′ CAGAGATGCAATTAACCCTCACTAAAGGGCGTAAGCTGTAAGTATG 3′
NRE 50–79 forward	T3	7177–7192	5′ CAGAGATGCAATTAACCCTCACTAAAGGGAGAATTCGTGTTGTTTGTTGTGT 3′

**Table 2:** Sequence of binding sites for hnRNP A1

Binding site	Sequence	Reference
hnRNP A1 consensus Selex high affinity binding site	UAGGGU	Burd and Dreyfuss (1994)
L1 ORF binding sites	CAGGG/AU	Zhao et al. (2004)
Putative LRE binding site	UUAGUGU	This manuscript
